# Supplementary material for: Sex-Induced Changes in Microbial Eukaryotes and Prokaryotes in Gastrointestinal Tract of Simmental Cattle
Source: Biology (Basel). 2024 Nov 15;13(11):932. doi: 10.3390/biology13110932 (PMC11591695; doi:10.3390/biology13110932)
Supplement: Supplementary file 1 [file biology-13-00932-s001.zip › Supplementary figure_Biology.pdf]

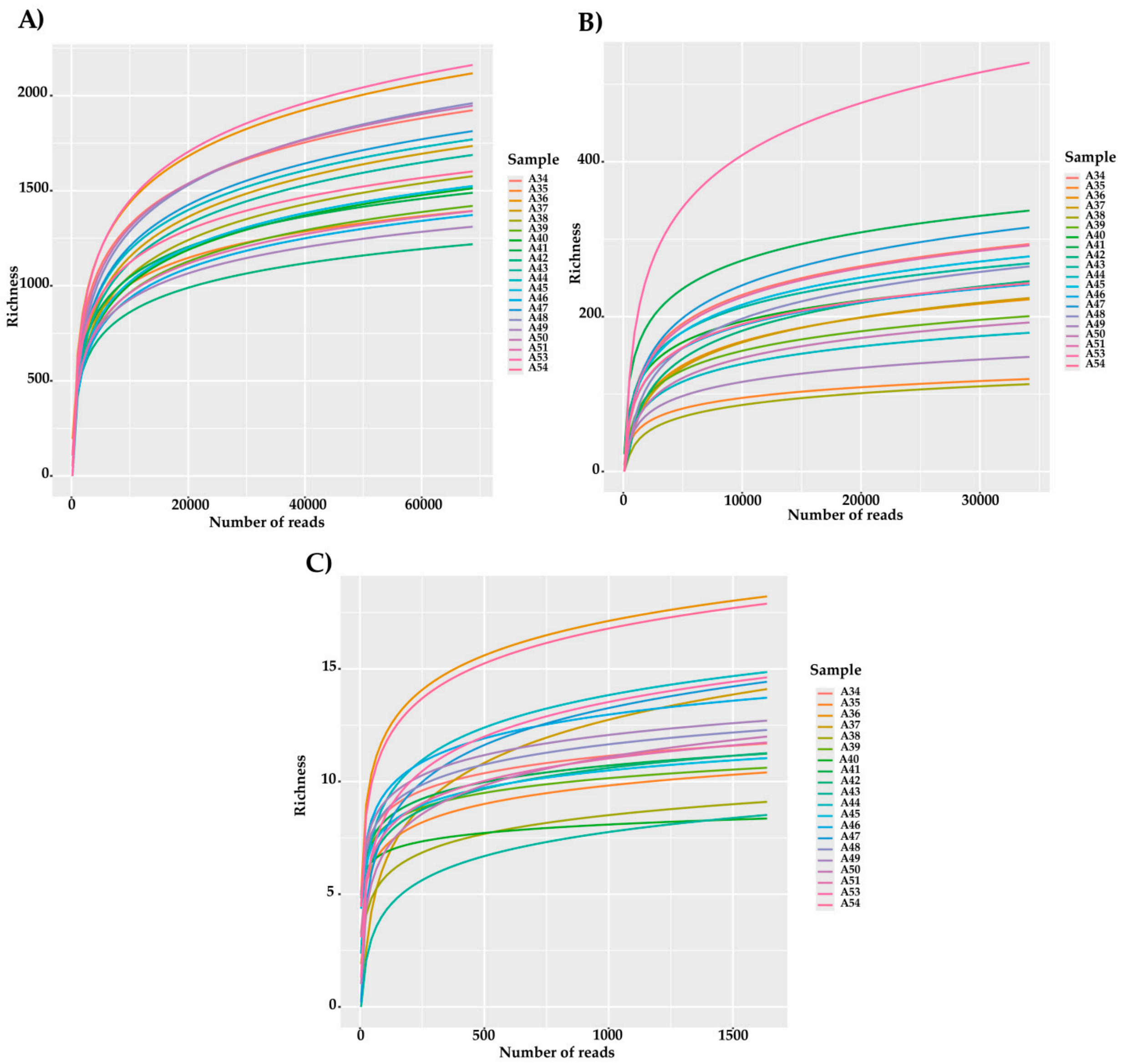

Figure S1: Species richness rarefaction curves. A) Species richness rarefaction curves show sequencing depth of 16S data obtained from bacteria from gut samples. B) Species richness rarefaction curves show sequencing depth of ITS data obtained from fungi from gut samples. C) Species richness rarefaction curves show sequencing depth of V4 data obtained from archaea from gut samples.

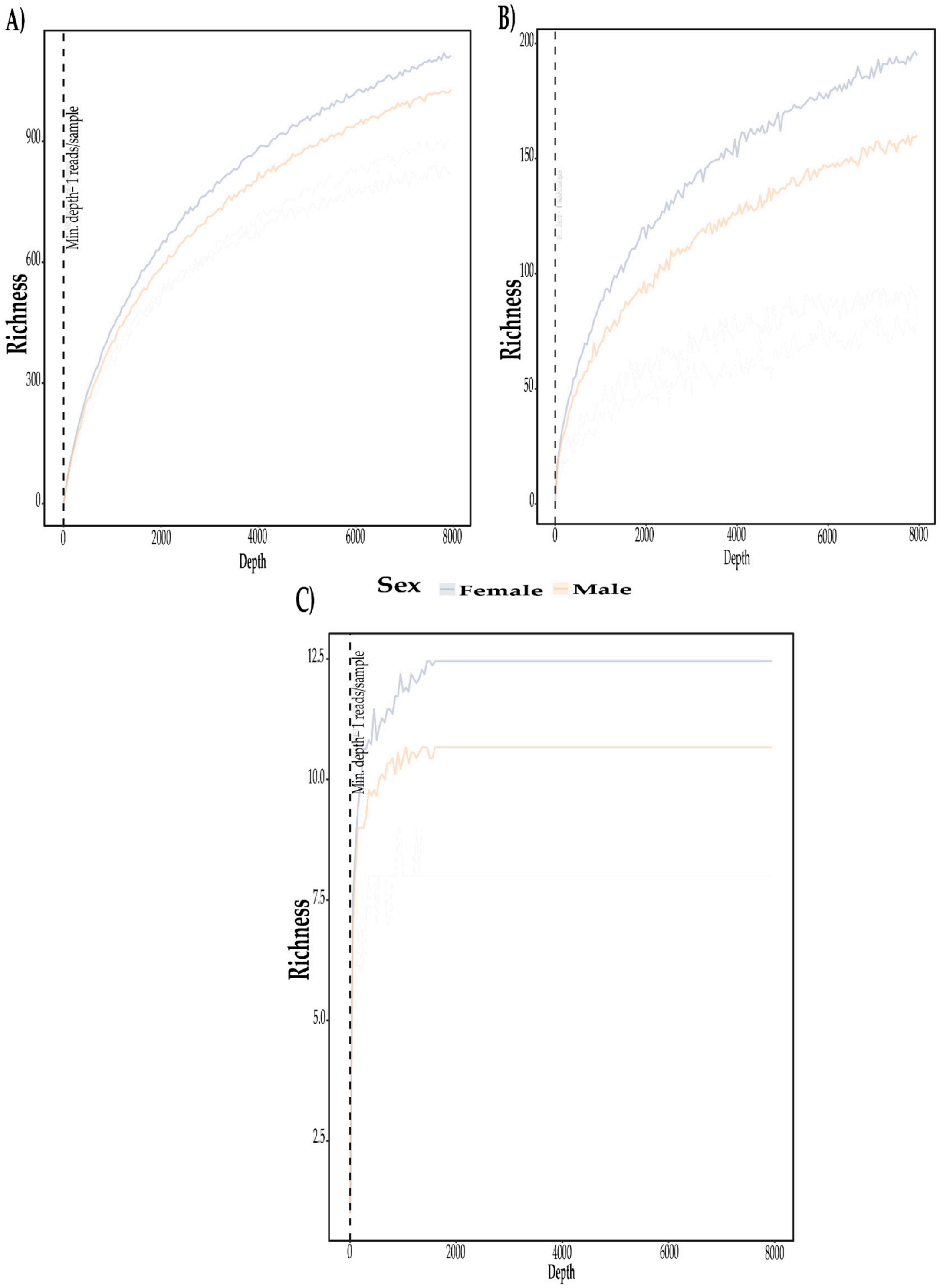

Figure S2: Species richness rarefaction curves for sex.

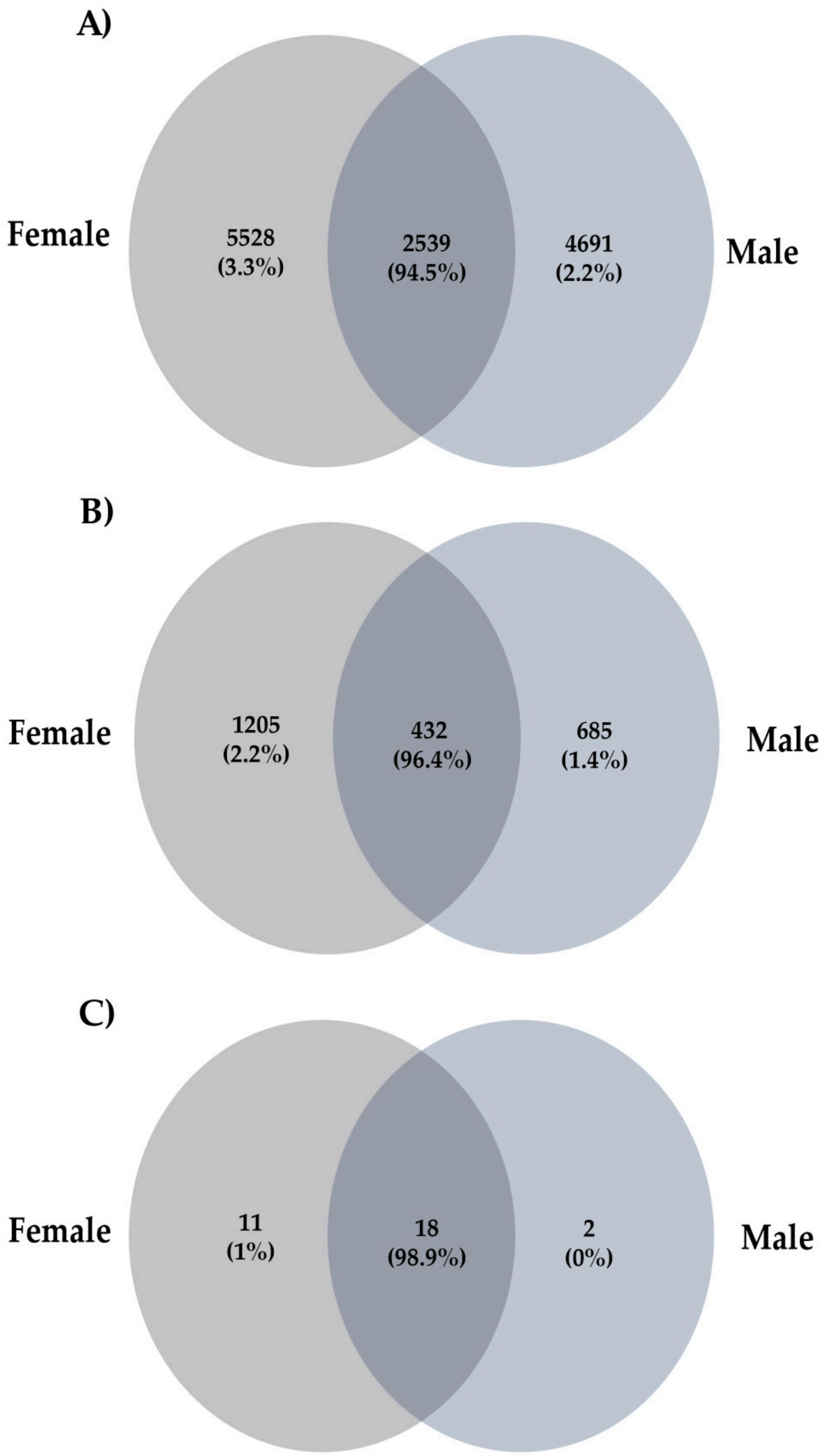

Figure S3: Venn diagrams presenting the shared and unique taxa among different sex groups of cattle. A) Bacteria Venn diagram. B) Fungal Venn diagram. C) Archaea Venn diagram

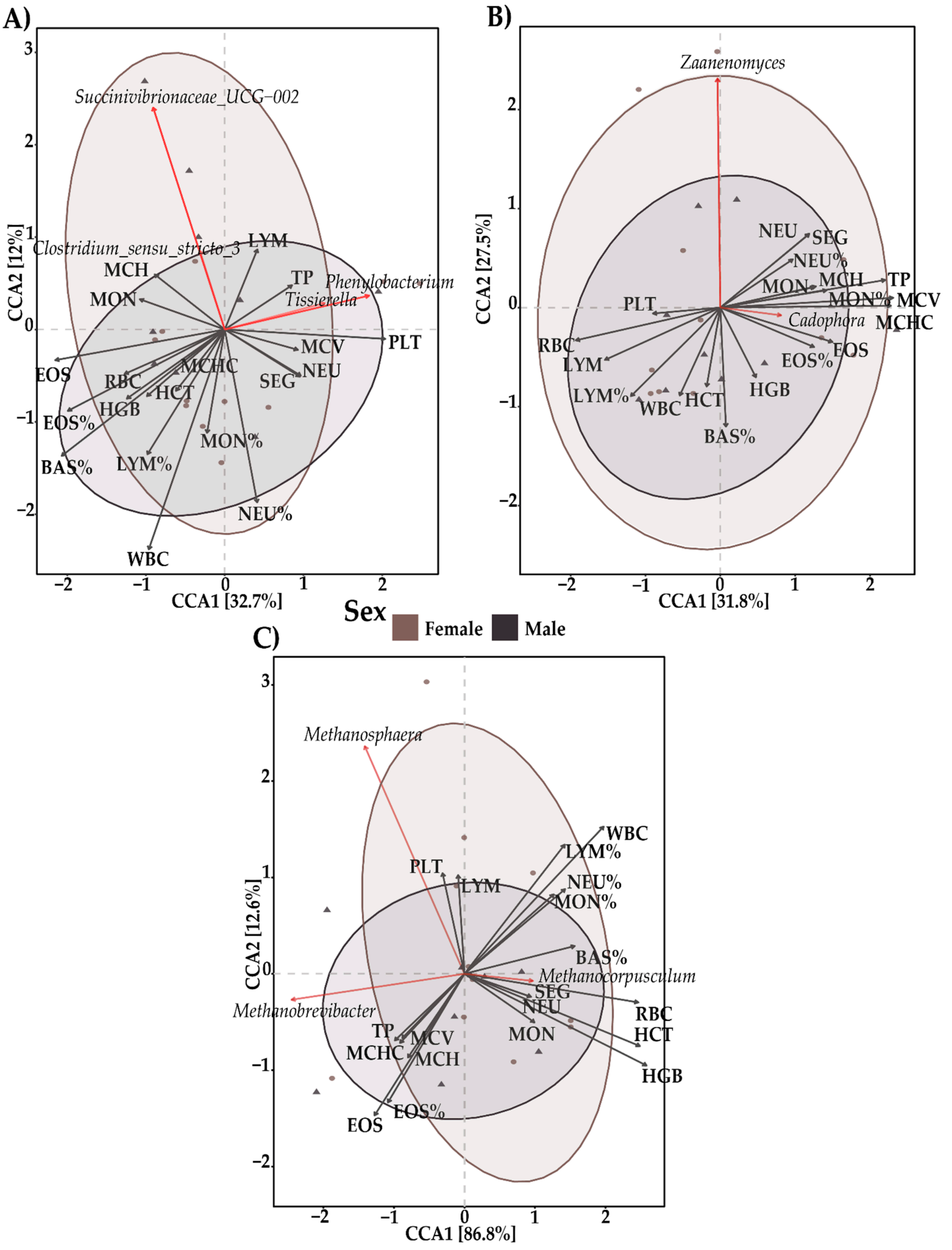

Figure S4: Canonical correlation analysis for Sex. A) Bacteria CCA. B) Fungal CCA. C) Archaea CCA

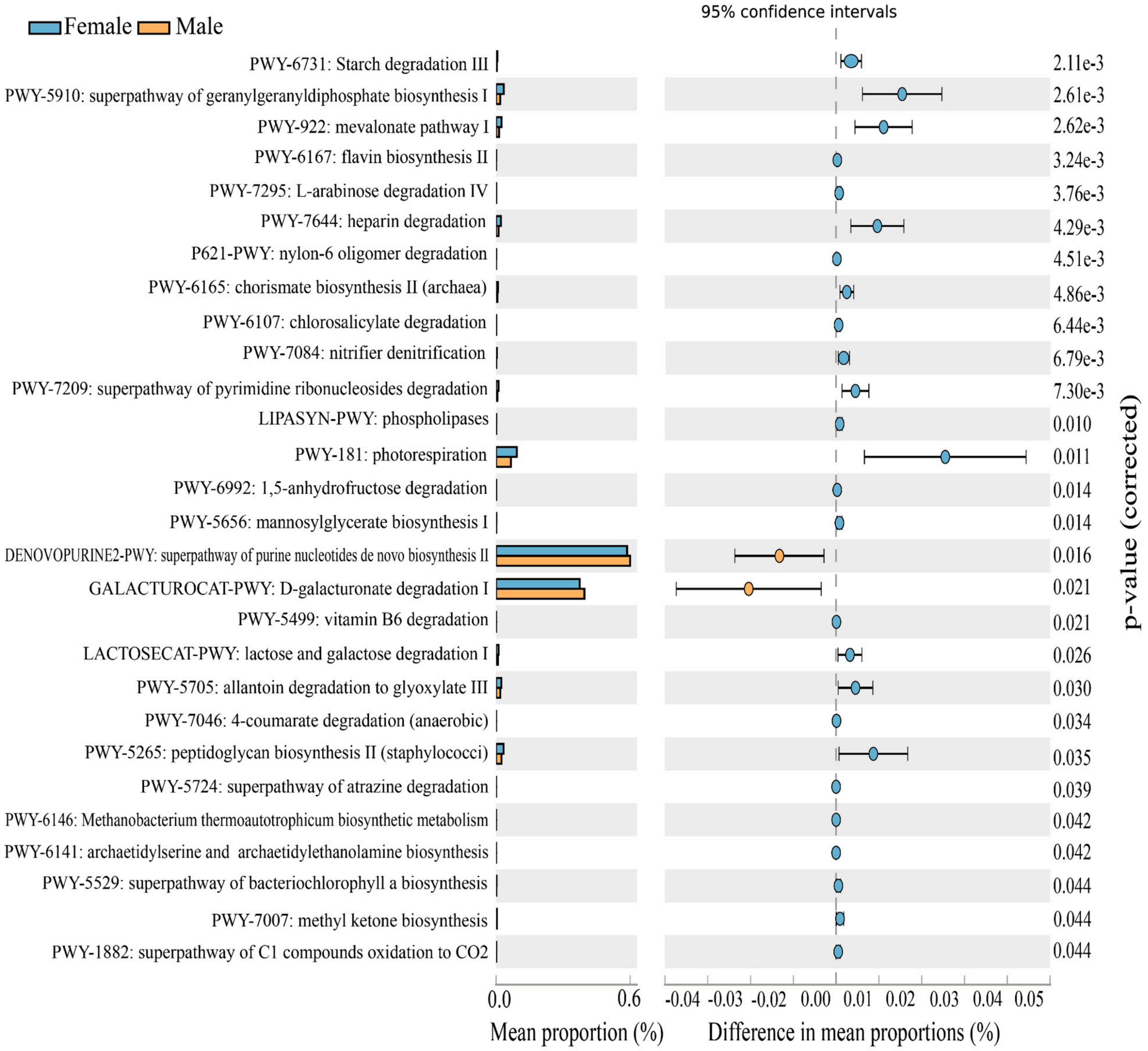

Figure S5: Prediction and comparison of bacterial community functions between sexes. PICRUSt2 was employed to infer changes in the composition of bacterial functional groups. STAMP software was utilized to assess the differences in KEGG functions between females and males, using Welch's two-sided t-test.
